# Multivariate time series analysis on the dynamic relationship between Class B notifiable diseases and gross domestic product (GDP) in China

**DOI:** 10.1038/s41598-016-0020-5

**Published:** 2016-12-23

**Authors:** Tao Zhang, Fei Yin, Ting Zhou, Xing-Yu Zhang, Xiao-Song Li

**Affiliations:** West China School of Public Health, Sichuan University, Chengdu, China

**Keywords:** Infectious diseases, Risk factors

## Abstract

The surveillance of infectious diseases is of great importance for disease control and prevention, and more attention should be paid to the Class B notifiable diseases in China. Meanwhile, according to the International Monetary Fund (IMF), the annual growth of Chinese gross domestic product (GDP) would decelerate below 7% after many years of soaring. Under such circumstances, this study aimed to answer what will happen to the incidence rates of infectious diseases in China if Chinese GDP growth remained below 7% in the next five years. Firstly, time plots and cross-correlation matrices were presented to illustrate the characteristics of data. Then, the multivariate time series (MTS) models were proposed to explore the dynamic relationship between incidence rates and GDP. Three kinds of MTS models, i.e., vector auto-regressive (VAR) model for original series, VAR model for differenced series and error-correction model (ECM), were considered in this study. The rank of error-correction term was taken as an indicator for model selection. Finally, our results suggested that four kinds of infectious diseases (epidemic hemorrhagic fever, pertussis, scarlet fever and syphilis) might need attention in China because their incidence rates have increased since the year 2010.

## Introduction

Accurate and timely surveillance of infectious diseases lays the foundation of effective disease control and prevention. To this end, China has built and kept improving its worldwide largest surveillance system for many decades. Currently, 39 notifiable infectious diseases are included in this system, classified as A, B and C according to their epidemic levels and potential population threats^1^. Both Class A (plague and cholera) and B notifiable diseases are with high risk of outbreak in rapid spread. In addition, most of the infectious diseases of Class B occur more frequently than those of the Class A, and cause more severe epidemics than those of the Class C. As a consequence, Class B infectious diseases are becoming the main focus of surveillance and their epidemic behaviours have been attracting more and more attention in recent years^2,3^.

Our previous work^3–6^ has provided an overview about the temporal trend of Class B notifiable diseases in China during the last decade, and it is natural to ask what the incidence will be in the coming years. Furthermore, since infectious diseases are not only medical but also social events, it is reasonable to involve social factors when making forecasts. Recently, one of the headline social news for China is that, according to the International Monetary Fund (IMF), the annual growth of Chinese GDP would decelerate below 7% after many years of soaring^7^. If this comes true, what will happen to the incidence rates of Class B notifiable diseases in China? In order to answer this question, the current paper will establish multivariate time series (MTS) models to study the dynamic relationship between Class B notifiable diseases and GDP.

To our knowledge, this is the first time to conduct such study in China both *dynamically* and *at the nationwide level*, though some interesting and inspiring researches have already been done. For example, Zhang and Jin^8^ investigated the relationship between the incidence of infectious diseases and economic growth in China in 2009. However, only Pearson correlation analyses were conducted in that study, which failed to reveal how historical data could dynamically predict the future incidence rates. On the contrary, our study employs both the vector auto-regressive (VAR) model and error-correction model (ECM) for multivariate time series analysis, which can effectively capture the dynamic interdependencies among multiple data sources. Besides, Tan, *et al.* ^9^ examined the county-level socio-demographic characteristics associated with syphilis and gonorrhoea in Guangdong Province by using linear and spatial lag regression, but considering China is a very large country with highly imbalanced development of regional economy, it is plausible to doubt whether the results are the same at the nationwide level.

**Figure 1:**
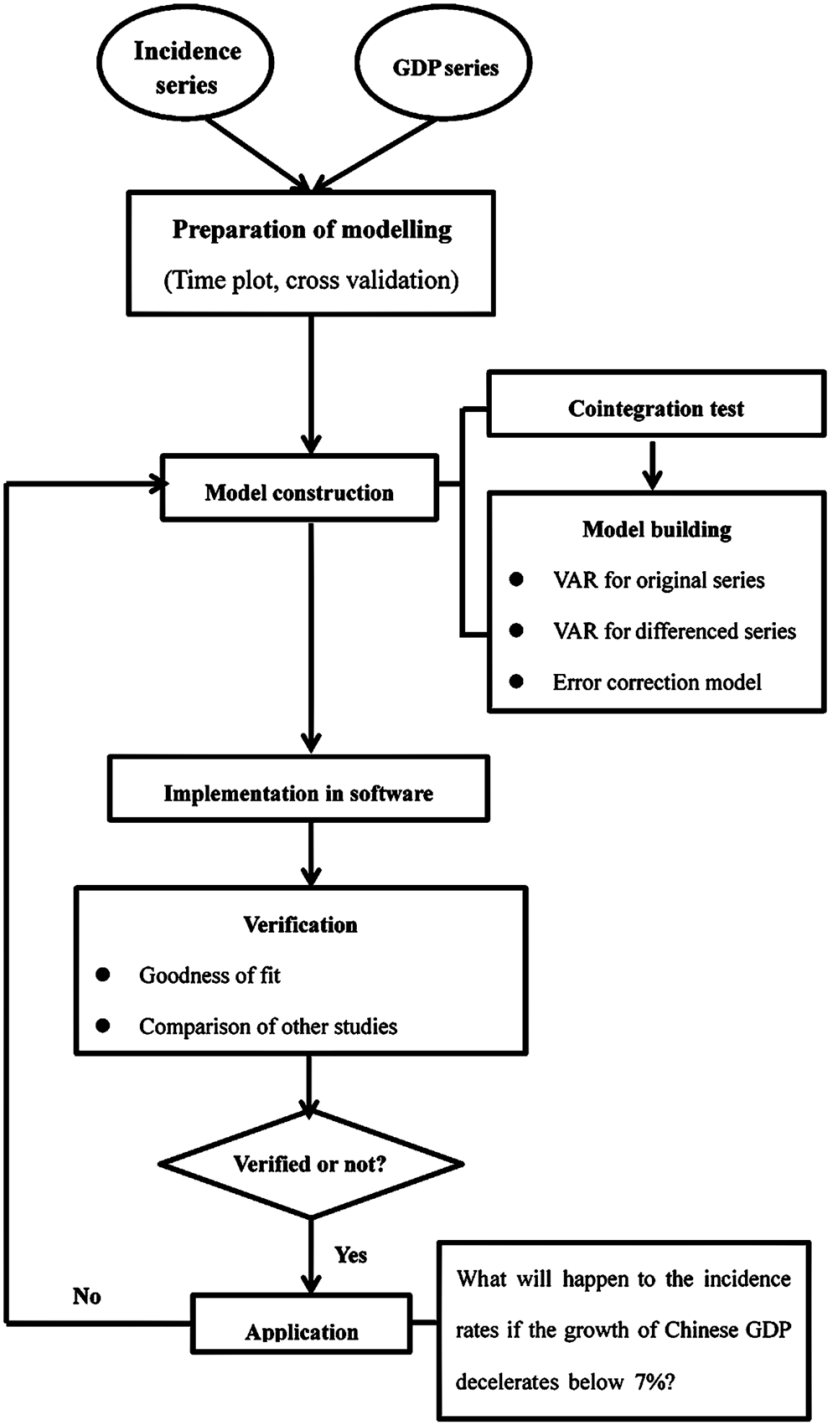
The process of modelling.

Figure 1 shows the flowchart of building MTS models between the infection and GDP time series data to forecast the future infection rates with the established model. To this end, the second part consists of the preparation, construction, implementation, verification and application of modelling. The third part reports the main results of this study. Finally, the last part ends the paper by concluding the new discoveries and future works to do in this research field.

## Materials and Methods

### The data

The data of infectious diseases came from China Health and Family Planning Statistical Yearbook (2015)^10^, ranging from 1978 to 2014. The GDP data of the corresponding period was obtained from the National Bureau of Statistics of China (http://data.stats.gov.cn/easyquery.htm?cn=C01). According to relevant laws and regulations, 26 types of infectious diseases were classified as Class B notifiable diseases in China, though three of them (severe acute respiratory syndromes, anthrax and human avian influenza) were actually treated as Class A notifiable diseases. In this study, 11 types of Class B notifiable diseases were included for analysis, and the rest diseases were excluded for the following reasons: ① the annual incidence rates remained constantly too low (usually < 0.05/10^5^) in the last decade, so it did not make much practical sense to explore their relationship with GDP (e.g., poliomyelitis and diphtheria); ② for the sake of scientific rigor, diseases with hard-to-interpret outliers were also excluded, e.g., measles; ③ the data of incidence for some diseases were not available until recent years, so their sample sizes were too small to build reliable statistical models, e.g., tuberculosis and dengue; ④ some endemic diseases prevailed only in certain areas, therefore it was not appropriate to analyse them at the nationwide level, e.g. brucellosis and schistosomiasis. Specifically, Table 1 listed the summary information of diseases to be analysed in this study.

**Table 1:** Class B infectious diseases.

Disease	Whether included in this study	Reasons for exclusion	Type of multivariate time series analysis model*
AIDS	No	Too short period	—
Virus hepatitis	Yes	—	ECM
Poliomyelitis	No	Too low rate	—
Severe acute respiratory syndromes	No	Treated as Class A notifiable diseases	—
Human avian influenza	No	Treated as Class A notifiable diseases	—
Measles	No	Outlier**	—
Epidemic hemorrhagic fever	Yes	—	VAR for original series
Rabies	Yes	—	VAR for original series
Epidemic encephalitis B	Yes	—	VAR for original series
Dengue	No	Too short period	—
Anthrax	No	Treated as Class A notifiable diseases	—
Bacterial and amebic dysentery	Yes	—	VAR for original series
Tuberculosis	No	Too short period	—
Typhoid fever	Yes	—	ECM
Pertussis	Yes	—	VAR for original series
Diphtheria	No	Too low rate	—
Epidemic cerebrospinal meningitis	No	Too low rate	—
Infantum tetanus	No	Too low rate	—
Scarlet fever	Yes	—	ECM
Brucellosis	No	Endemic disease	—
Gonorrhoea	Yes	—	VAR for original series
Syphilis	Yes	—	VAR for differenced series
Leptospirosis	No	Too low rate	—
Schistosomiasis	No	Endemic disease	—
Malaria	Yes	—	VAR for differenced series
Influenza A(H_1_N_1_)/H_7_N_9_ avian influenza***	No	Task adjustment	—

*Three types of multivariate time series analysis models were used in this study, that is, the VAR for original series, VAR for differenced series and the ECM, see more information in the rest of paper. **The incidence rate of measles was zero in 2003, but was far great than zero in 2002 and 2004. This study did not find reasonable explanation for this outlier, so measles was not included for analysis. ***According to regulation of government, influenza A (H_1_N_1_) has been adjusted from Class B to Class C notifiable disease since 2014, while H_7_N_9_ avian influenza was included as Class B since 2013 (http://www.nhfpc.gov.cn/jkj/s3577/201311/f6ee56b5508a4295a8d552ca5f0f5edd.shtml).

### The preparation of modelling

Before modelling, the ***time plot*** and ***cross-correlation matrices*** ^11^ were applied to illustrate the characteristics of data and help select the appropriate analysis models. The time plot showed the data against the time index (i.e. incidence *v.s*. year, or GDP *v.s*. year), and could present temporal characteristics such as short-term oscillation and long-term trend. Additionally, considering the multivariate cases, the cross-correlation matrices were also used to describe the dynamic relationships. For example, GDP in recent years may be correlated with the incidence rate of infectious disease in the coming years. In view of this, let {*x*_1,*t*_} and {*x*_2,*t*_} denote the value of incidence rate and GDP at year *t*, respectively. Then the whole data observed at year *t* could be noted as ***x***_***t***_ = {*x*_1,*t*_, *x*_2,*t*_}, where ***x***_***t***_ was a vector with two ***series components*** (in this paper, boldface notation was used to indicate vectors and matrices). For any time lag *k* (*k* is an integer), the lag-*k* cross-correlation matrix was defined as *ρ*_*ij*_(*k*), which was the correlation coefficient between *x*_*i*,*t*_ and *x*_*j*,*t+k*_ (*i*, *j* = 1, 2). For illustration, if both *i* = *j* = 1, then *ρ*_11_(*k*) measured the correlation of incidence rates between the current year and *k* year ahead (if *k* < 0) or later (if *k* > 0); likewise, if *i* = 1, *j* = 2, and *k* > 0, then *ρ*_12_(*k*) was the dependence of current incidence on the GDP at *k* year later. In this way, it not only considered temporal effect, but also accounted for the correlation between different series components.

### Model construction

To guarantee the fitted and forecasted incidence rates were non-negative, all the data were logarithmically transformed to **ln** ***x***_***t***_ = {ln*x*_1,*t*_, ln*x*_2,*t*_} before modelling. Then the MTS model was built based on **ln** ***x***_***t***_, and this model was further employed to make forecasts. Finally, the inverse-logarithmic (or exponential) transformation was taken on the fitted and forecasted results to transform them back into original form.

As mentioned above, both VAR and ECM are useful models for multivariate time series analysis, but each of them has its own applicable conditions. Tsay^11^ proposed a two-step testing procedure to help select a most appropriate model. The first step is to build an ECM for the vector series **ln** ***x***_***t***_:

1 $$\nabla {\bf{ln}}{{\boldsymbol{x}}}_{{\boldsymbol{t}}}={{\boldsymbol{\mu }}}_{{\boldsymbol{t}}}+{\boldsymbol{\Pi }}\,{\bf{ln}}{{\boldsymbol{x}}}_{{\boldsymbol{t}}{\boldsymbol{-}}{\bf{1}}}+{{\boldsymbol{\phi }}}_{1}\nabla {\bf{ln}}{{\boldsymbol{x}}}_{{\boldsymbol{t}}{\boldsymbol{-}}{\bf{1}}}+\cdots +{{\boldsymbol{\phi }}}_{{\bf{p}}-1}\nabla {\bf{ln}}{{\boldsymbol{x}}}_{{\boldsymbol{t}}{\boldsymbol{-}}{\boldsymbol{p}}{\rm{+}}{\bf{1}}}+{{\boldsymbol{a}}}_{{\boldsymbol{t}}},$$

where $\nabla {\bf{ln}}{{\boldsymbol{x}}}_{{\boldsymbol{t}}}$ is the differenced series of ${\bf{ln}}{{\boldsymbol{x}}}_{{\boldsymbol{t}}}(\nabla {\bf{ln}}{{\boldsymbol{x}}}_{{\boldsymbol{t}}}=\,{\bf{ln}}{{\boldsymbol{x}}}_{{\boldsymbol{t}}}-\,{\bf{ln}}{{\boldsymbol{x}}}_{{\boldsymbol{t}}{\boldsymbol{-}}{\bf{1}}})$, ***a***_*t*_ is the residual series, and matrix **II** is called *error-correction term*. Then according to the testing result on rank **II**, three types of MTS models are utilised, i.e., the VAR model for original series {**ln** ***x***_***t***_}, the VAR model for differenced series $\{\nabla {\bf{ln}}{{\boldsymbol{x}}}_{{\boldsymbol{t}}}\}$ and the ECM, below are some more details.

1.  VAR model for original series.
  If rank(**II**) = 2, it implies the ECM is not so informative that the VAR model could analyse **ln** ***x***_***t***_ directly. The VAR model is an extension of traditional autoregressive (AR) model from univariate to multivariate time series analysis. It reflects the influence of the last *p* historical data on the current one, which can be written as
  2 $${\bf{ln}}{{\boldsymbol{x}}}_{{\boldsymbol{t}}}={{\boldsymbol{\mu }}}_{{\boldsymbol{t}}}+{{\boldsymbol{\phi }}}_{{\bf{1}}}^{\ast }\,{\bf{ln}}{{\boldsymbol{x}}}_{t{\boldsymbol{-}}{\bf{1}}}+\cdots +{{\boldsymbol{\phi }}}_{{\boldsymbol{p}}-1}^{\ast }\,{\bf{ln}}{{\boldsymbol{x}}}_{{\boldsymbol{t}}{\boldsymbol{-}}{\boldsymbol{p}}{\rm{+}}{\bf{1}}}+{{\boldsymbol{a}}}_{{\boldsymbol{t}}}.$$
2.  VAR model for differenced series.
  When the testing result is null, i.e., rank(**II**) = 0, it indicates that the dynamic relationship between incidence and GDP are nonstationary, and the ***differencing*** technique will be used to transform it into stationary one. Consequently, the VAR(*p*) model would be applied to the differenced series $\nabla {\rm{ln}}{{\boldsymbol{x}}}_{{\boldsymbol{t}}}$ instead of **ln** ***x***_***t***_, that is,
  3 $$\nabla {\bf{ln}}{{\boldsymbol{x}}}_{{\boldsymbol{t}}}={{\boldsymbol{\mu }}}_{{\boldsymbol{t}}}+{{\boldsymbol{\phi }}}_{{\bf{1}}}\nabla {\bf{ln}}{{\boldsymbol{x}}}_{{\boldsymbol{t}}{\boldsymbol{-}}{\bf{1}}}+\cdots +{{\boldsymbol{\phi }}}_{{\boldsymbol{p}}-{\bf{1}}}\nabla {\bf{ln}}{{\boldsymbol{x}}}_{{\boldsymbol{t}}{\boldsymbol{-}}{\boldsymbol{p}}+{\bf{1}}}+{{\boldsymbol{a}}}_{{\boldsymbol{t}}}.$$
3.  ECM.

The ECM is applied when rank(**II**) = 1, which is of the form Eq. (1). The ECM could be considered as a supplement to the VAR model by adding an error-correction term to the latter. Generally, the VAR model characterises the long-term trend, while the error-correction term adjusts the short-term oscillation.

### The implementation of modelling in R

In this study, the VAR and ECM were estimated by ordinary least squares (OLS) method. To determine the unknown order *p* for the model, the Akaike information criterion (AIC)^12^ would be used. All statistical analyses were performed in R 3.2.3 (the R Foundation for Statistical Computing)^13^, a free software environment for statistical computing and graphics. Computing packages {vars}^14^ and {tsDyn}^15^ can be downloaded from the Comprehensive R Archive Network (CRAN) at http://cran.r-project.org/ and installed in advance. The cross-correlation matrices could be calculated by the command *ccf*, and the VAR and ECM could be estimated by the command *VAR* and *VECM*, respectively.

### The verification of modelling

Since the model was built for the aim of forecasting, verification was considered to make the results more convincing. In particular, models were verified in three ways: ① the goodness-of-fit; ② the comparison with other models; ③ and with previous studies.

In this study, the goodness-of-fit consisted of two measures to evaluate the fitting performance of the model. One was the *mean squared percentage error* (MSPE), which quantified the difference between the fitted incidence rates and the real ones. The other one was the Ljung-Box test for the residuals {***a***_***t***_}, which was to test whether the model was good enough to efficiently extract useful information from the data and thus leave the residuals to be white noise (with zero mean and constant standard deviation).

The second way for verification was to compare the results of our approaches with those of traditional method. Since the autoregressive integrated moving average (ARIMA) model has been one of the most widely used techniques^6^, it served as benchmark to evaluate the performance of MTS model in this study.

The third way for verification was to compare our results with some similar previous studies. As mentioned in the Introduction part, since the relationship between incidence rate and GDP has been previously studied to some degree, it could help us to verify whether our new results make practical sense or not.

### The application of modelling

After the models were built and verified, they could finally be utilised to make forecasts on future incidence rates if the growth of Chinese GDP remained below 7%. To make the forecasting step clear, we took the ECM as an illustration, which was almost the same for VAR model. The ECM model represented by Eq. (1) could be rewritten for incidence (*x*_1,*t*_) and GDP (*x*_2,*t*_) series, respectively, that was

$$(\begin{array}{c}\nabla \mathrm{ln}{x}_{1,t}\\ \nabla \mathrm{ln}{x}_{2,t}\end{array})=(\begin{array}{c}{\mu }_{1,t}\\ {\mu }_{2,t}\end{array})+(\begin{array}{cc}{\pi }_{11} & {\pi }_{12}\\ {\pi }_{21} & {\pi }_{22}\end{array})(\begin{array}{c}\mathrm{ln}{x}_{1,t-1}\\ \mathrm{ln}{x}_{2,t-1}\end{array})+\sum _{i=1}^{p}(\begin{array}{cc}{\phi }_{i,11} & {\phi }_{i,12}\\ {\phi }_{i,21} & {\phi }_{i,22}\end{array})(\begin{array}{c}\nabla \mathrm{ln}{x}_{1,t-i}\\ \nabla \mathrm{ln}{x}_{2,t-i}\end{array})+(\begin{array}{c}{a}_{1,t}\\ {a}_{2,t}\end{array}).$$

Then at the current time point *t*, the future incidence at time point *t* + *l* (*l* ≥ 1) could be forecasted as

4 $$\nabla \mathrm{ln}{\tilde{x}}_{1,t+l}={\mu }_{1,t}+{\pi }_{11}\,\mathrm{ln}{\tilde{x}}_{1,t+l-1}+{\pi }_{12}\,\mathrm{ln}\,{\tilde{x}}_{2,t+l-1}+\sum _{i=1}^{p}({\phi }_{i,11}\nabla \mathrm{ln}{\tilde{x}}_{1,t+l-i}+{\phi }_{i,12}\nabla \mathrm{ln}{\tilde{x}}_{2,t+l-i})+{a}_{1,t+l}.$$

For Eq. (4), if *l* − *i *< 0, then $\nabla \mathrm{ln}\,{\tilde{x}}_{1,t+l-i}$ and $\nabla \mathrm{ln}\,{\tilde{x}}_{2,t+l-i}$ were the historical data which were already known; on the other hand, if *l* − *i *> 0, they represented future unknown values. In this case, the future incidence-related information $\nabla \mathrm{ln}\,{\tilde{x}}_{1,t+l-i}$ (*l* − *i *> 0) would be calculated recursively through Eq. (4), and the future data of GDP could be directly referred from the IMF website^7^, where future values of Chinese GDP till 2020 were predicted and listed.

## Results

### The results of modelling preparation

**Figure 2:**
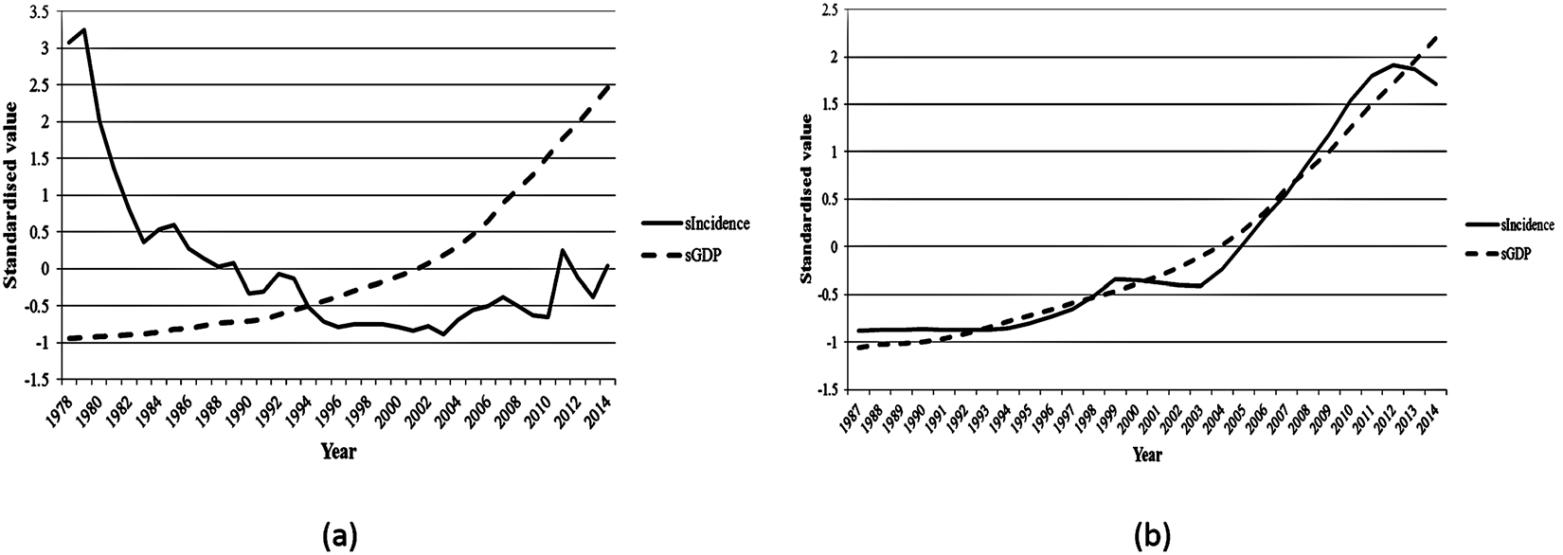
(**a**) The time plot of the standardised scarlet fever incidence and GDP; (**b**) The time plot of the standardised syphilis incidence and GDP.

Since incidence and GDP series differed dramatically in scale, to better illustrate their mutual relationship, each series were standardised before time plotting. The relationships illustrated by the time plots could be summarised into two categories. The first category, as was shown by Fig. 2(a), indicated that the incidence rate fell dramatically as GDP increased. In contrast, Fig. 2(b) presented the second category, suggesting both incidence rate and GDP were increasing. Consequently, cross-correlation matrices were applied to further identify the direction of relationship, which further classified the diseases into the ***positively-correlated group*** (gonorrhoea and syphilis) and the ***negatively-correlated group*** (epidemic hemorrhagic fever, malaria, pertussis, rabies, bacterial and amoebic dysentery, epidemic encephalitis B, scarlet fever, typhoid fever and virus hepatitis).

### The model

The model types selected for each disease were shown in Table 1. For better understanding, syphilis, epidemic hemorrhagic fever and typhoid fever were taken as examples for each type. Meanwhile, in order to keep the model as parsimonious as possible, only those statistically significant estimated coefficients were included in the model.

1.  VAR model for epidemic hemorrhagic fever.
  For the log-transformed incidence,
  $$\mathrm{ln}\,{x}_{1,t}=3.123+1.054\,\mathrm{ln}\,{x}_{1,t-1}-0.333\,\mathrm{ln}\,{x}_{1,t-2}-0.247\,\mathrm{ln}\,{x}_{2,t-2},$$
  and for the log-transformed GDP,
  $$\mathrm{ln}\,{x}_{2,t}=0.008\,\mathrm{ln}\,{x}_{1,t-1}+1.766\,\mathrm{ln}\,{x}_{2,t-1}-1.210\,\mathrm{ln}\,{x}_{2,t-2}+0.449\,\mathrm{ln}\,{x}_{2,t-3}.$$
2.  VAR model for differenced syphilis.
  For the differenced log-transformed incidence,
  $$\nabla \mathrm{ln}{x}_{1,t}=-0.450+0.546\nabla \mathrm{ln}\,{x}_{1,t-1}+5.795\nabla \mathrm{ln}{x}_{2,t-1},$$
  and for the differenced log-transformed GDP,
  $$\nabla \mathrm{ln}\,{x}_{2,t}=0.038+0.577\nabla \mathrm{ln}\,{x}_{2,t-1}.$$
3. ECM for typhoid fever.

In the analysis of typhoid fever, a trend term “*t*” was included in the model to account for long-term trend. For the differenced log-transformed incidence,

$$\begin{array}{rcl}\nabla \mathrm{ln}\,{x}_{1,t} & = & 0.431-0.020t-0.176\mathrm{ln}\,{x}_{1,t-1}+0.022\mathrm{ln}\,{x}_{2,t-1}\\  &  & +\,0.328\nabla \mathrm{ln}\,{x}_{1,t-1}-3.672\nabla \mathrm{ln}{x}_{2,t-1}+3.502\nabla \mathrm{ln}\,{x}_{2,t-2},\end{array}$$

and for the differenced log-transformed GDP,

$$\nabla \mathrm{ln}\,{x}_{2,t}=0.077+0.672\nabla \mathrm{ln}\,{x}_{2,t-1}-0.048\nabla \mathrm{ln}\,{x}_{1,t-2}-0.527\nabla \mathrm{ln}\,{x}_{2,t-2}.$$

Overall, from the above three examples, it could be seen that both the historical incidence rates and GDP would affect the current incidence rate. However, on the other hand, incidence rate scarcely had any influence on GDP. Therefore, the results suggested there was *unidirectional* relationship from GDP to the incidence rates of the eleven Class B notifiable infectious diseases included in this study for the last three decades.

### Model interpretation

The influence of GDP on disease incidence could be in either positive or negative way. On the one hand, with the increase of GDP in China, the expenditure on health and medicine has been enhanced. To name but a few examples, the government expenditure on health has annually risen by 18.66% on average since the year of 1990^10^. Besides, the number of people benefitting from the water-improving project accelerated from 0.6 billion in 1990 to 0.9 billion in 2014. Those events were undoubtedly of great benefit to disease control and prevention. On the other hand, this study also found that the incidence rates of gonorrhoea and syphilis had risen along with the economy development. Although the reasons were various, it was at least plausible to say that the increasing power of purchase made some people more financially affordable to extramarital and premarital sex behaviours, which gave rise to the risks of sexually transmitted infections^6,16^.

The absence of influence from incidence rates to GDP might seem implausible at the first sight, but it would be easier to understand if the following three points were taken into consideration. At first, GDP means a monetary measure of the market value of all final goods and services produced in a period^17^, and the influence of incidence on GDP should be distinguished from the money of loss due to diseases. For example, suppose that 1,000 dollars were paid by a patient for medicine and health care services, it indeed caused a financial loss for the patient, but this amount of money was also counted as the market value produced by the medical and health industry. Therefore, from the economic point of view, the payment was a promotion instead of poison for GDP. Secondly, as told by the widely accepted Cobb-Douglas production function^18^, diseases may deteriorate GDP through damaging the health of labours, which was generally measured by the disability adjusted of life years (DALYs). However, according to the Global Burden of Disease Study 2013 (GBD 2013)^19^, not only were infectious diseases no longer the main causes of DALYs in China, but also the DALYs of infectious diseases had all declined globally ever since 1990. Finally, it should be reminded that this study merely included 11 types of Class B infectious diseases in China. Although some other researchers have declared infectious diseases had an impact on economics^20^, what they mainly referred were those diseases lack of timely diagnosis and prevention. Since the diseases in our study were under well prevention, treatment and control in China, it was appropriate to judge that there were no essential contradictions between this study and others.

### The verification result

**Figure 3:**
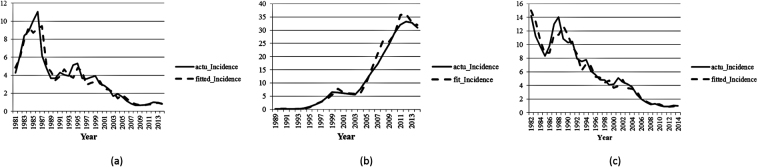
(**a**) The fitting plot for epidemic hemorrhagic fever; (**b**) The fitting plot for syphilis; (**c**) The fitting plot for typhoid fever.

The fitting plots for epidemic hemorrhagic fever, syphilis and typhoid fever were shown in Fig. 3, which illustrated that the fitted incidence rates were generally in consistent with the actual ones. The fitting plots for other diseases were the same, and thus not presented here due to the limited space.

Table 2 presented the overall evaluation results on goodness-of-fit. For each disease, the second column in Table 2 presented the Ljung-Box test result of MTS model, indicating this model was efficient enough to extract information from data. Besides, Table 2 also listed results for the comparison of MSPEs between the MTS and ARIMA model. It could be seen from column 3 and 4 that the MSPEs of MTS model were very small (<0.04 on average), and even smaller than those of the ARIMA model. Although the MSPEs of the fitted incidence rates for epidemic encephalitis B, malaria, rabies and scarlet fever were slightly bigger, however, after careful check of the original data, we found the inconsistencies were mainly occurred in the early 1980s, so it was plausible to infer that these inconsistencies would not jeopardise the validity of forecasts.

**Table 2:** The goodness-of-fit results of MTS and ARIMA model for each disease.

Disease	*P* value of Ljung-Box test for MTS model residuals*	MSPE of MTS model	MSPE of ARIMA model
Bacterial and amebic dysentery	0.2687	0.012967	0.0543
Epidemic encephalitis B	0.9867	0.038293	0.0741
Epidemic hemorrhagic fever	0.9009	0.029741	0.0355
Gonorrhoea	0.9072	0.018547	0.0403
Malaria	0.8817	0.041325	0.0402
Pertussis	0.6865	0.032576	0.0439
Rabies	0.8484	0.045806	0.0543
Scarlet fever	0.6995	0.041423	0.0497
Syphilis	0.5432	0.036579	0.0447
Typhoid fever	0.3546	0.020911	0.0336
Virus hepatitis	0.6839	0.015540	0.0166

*The significance level *α* was set to be 0.05 in advance. The null hypothesis of Ljung-Box test declared that the testing series to be white noise, thus it was reasonable to say the model was good at fitting if such null hypothesis for its corresponding residual series could not be rejected.

Another approach to verify the result of modelling was to compare our results with those of other studies. As has been mentioned above, the findings of correlation between GDP and infectious diseases have coincided with most previous studies^2,3,9,21,22^, but with only a few exceptions^9,23–25^. In those exceptional studies, GDP/GDP per capita was not significant predictor of the infectious diseases (e.g., syphilis, gonorrhoea, malaria and Hepatitis C), however, they still displayed the same signs of correlation as this study did. Meanwhile, from the perspective of epidemiology and biostatistics, certain variables should be remained even though they had non-significant effects, because of the logical importance in the particular problem^26^. Therefore, it was plausible to say that the results of this study did not essentially contradict with those of previous researches.

In addition, some concerns may arise over the matter of spatial stratified heterogeneity, that is, whether the relationship between incidence and GDP distributed unevenly across different parts of the whole country. To this end, this study utilised the *q*-statistics proposed by Wang *et al.* ^27^ to make hypothesis test. According to the National Bureau of Statistics of China, the 31 provinces in mainland China were classified into eastern, middle and western regions, respectively. For each disease, the testing results were shown in Table 3, which suggested that the null hypothesis *H*_0_ (i.e., no spatial stratified heterogeneity) could not be rejected yet.

**Table 3:** The testing results of spatial stratified heterogeneity for each disease.

Disease	*F*	*f*_1_	*f*_2_	lambda	cutoff point	*P*
Bacterial and amoebic dysentery	3.151833	2	28	0.351702	3.9048	>0.05
Epidemic encephalitis B	0.942817	2	28	0.456857	4.0651	>0.05
Epidemic hemorrhagic fever	0.142923	2	28	0.054094	3.4302	>0.05
Gonorrhoea	0.517519	2	28	0.559956	4.2188	>0.05
Malaria	0.229567	2	28	0.048982	3.4217	>0.05
Pertussis	3.138548	2	28	0.599739	4.2772	>0.05
Rabies	2.768148	2	27	0.580805	4.2672	>0.05
Scarlet fever	2.535921	2	28	0.810873	4.5799	>0.05
Syphilis	1.584857	2	20	1.73419	6.0807	>0.05
Typhoid fever	0.461945	2	20	2.97123	7.6324	>0.05
Virus hepatitis	0.817709	2	28	0.180542	3.6358	>0.05

*The significance level *α* was set to be 0.05 in advance. The *F-*statistics (column 2) was constructed based on the *q*-statistics, which followed a non-central *F*-distribution, with first and second degree of freedom *f*_1_ (column 3) and *f*_2_ (column 4), and noncentrality parameter lambda (column 5). Column 6 listed the 95% cutoff point, and by comparing it with the *F* statistics, it could be inferred whether *P* > *α* or not (column 7).

### The application of model

After verification, the corresponding MTS models were utilised to forecast the incidence rates of the next five years. Table 4 provided the changing incidence rates for each disease from 1978 to 2020. The incidence rates from 1978 to 2014 were observed ones, and those from 2015 to 2020 were predicted from the model. Except for scarlet fever, the incidence rates of all the other diseases were expected to decrease between 2015 and 2020. If the forecasting results were true, then it meant the incidence rate of scarlet fever would have been increasing ever since 2000. Besides, Table 4 also indicated that the incidence rates of epidemic hemorrhagic fever, pertussis and syphilis had risen to some degree during the last decade. These results raised warnings for future disease outbreaks, which were further discussed in the next part.

**Table 4:** The change rate for each disease from 1978 to 2020.

Disease	1978~1989*	1990~1999	2000~2009	2010~2014	2015~2020
Bacterial and amoebic dysentery	−0.80	−0.62	−0.50	−0.40	−0.21
Epidemic encephalitis B	−0.70	−0.80	−0.69	−0.68	−0.53
Epidemic hemorrhagic fever	1.32	0.07	−0.78	0.17	−0.33
Gonorrhoea**	495.50	1.77	−0.60	−0.13	−0.27
Malaria	−0.96	−0.77	−0.48	−0.60	−0.51
Pertussis	−0.98	−0.72	−0.74	0.92	−0.32
Rabies	0.88	−0.91	3.25	−0.53	−0.87
Scarlet fever	−0.72	−0.54	0.54	1.56	0.83
Syphilis***	1.25	27.26	2.84	0.07	−0.59
Typhoid fever	−0.30	−0.60	−0.69	−0.03	−0.33
Virus hepatitis	0.22	−0.39	0.65	−0.09	−0.25

*For each column, the change rate = (the incidence of the last year-the incidence of first year)/the incidence of first year. **The first period for gonorrhoea was from 1981 to 1989. ***The first period for syphilis was from 1987 to 1989.

## Discussion

In this study, a new approach based on MTS model was provided to investigate not only the direction of dynamic relationship between incidence rates of Class B notifiable diseases and GDP in China, but also the effect size of this relationship. Statistically significant evidence was found that the Chinese GDP growth affected its incidence rates of Class B notifiable diseases over the past thirty years. In addition, based on the IMF’s forecasts about future Chinese GDP, our models forecasted the future trends of incidence rates in the next five years, and therefore indicated the key point of disease control and prevention from our own view. Finally, these results have been verified in multiple ways to increase their creditability.

This study highlighted the importance and necessity of merging multiple sources of information into the surveillance of infectious diseases. At least two kinds of information were proved useful by previous studies^28,29^: historical incidence data and exogenous variables including but not limited to GDP. Therefore, this study built MTS models to account for both of them. It could bring benefits in three ways: ① characterising both long-term and short-term relationships between incidence rate and GDP; ② making conditional predictions; ③ reducing uncertainty by introducing extra information. Our results directly supported the first two of them. As for the last one, since this study has already demonstrated cross-correlation between incidence rate and GDP series, it was plausible to confirm it from the view of information theory.

Another feature of this study was the provision of integrative approaches for multivariate time series analysis of infectious diseases. To account for any possible relationship between incidence rate and GDP series, totally three kinds of MTS models were considered: VAR model for original series, VAR model for differenced series and ECM. As for a certain infectious disease, based on the rank of error-correction term, clear indication was given on which of those models should be taken for analysis. Furthermore, this study has provided the R software codes to realise the whole modelling procedures. It was highly expected that all these attempts would encourage and help practioners to apply our methods to study the relationship between incidence rates of infectious disease with many other factors besides GDP.

It is quite necessary to emphasise that the ultimate goal of disease surveillance is to suggest what should be done in the future rather than to make mere forecasts. This study contributes to this goal by warning four kinds of infectious diseases (epidemic hemorrhagic fever, pertussis, scarlet fever and syphilis) might need special attention because their incidence rates have increased since the year 2010. Epidemic hemorrhagic fever is caused by hanta viruses, and its incidence rate is positively correlated with rodent density^30^, so rodent control and extinguishment needs to be strengthened. Since pertussis is vaccine-preventable disease, future work needs be done to maintain high level of DPT (diphtheria, tetanus and pertussis combined vaccine) immunisation coverage. In recent years, scarlet fever mostly occurred among school children, however, there has not been any efficient vaccine for prevention yet; therefore, it is imperative to protect susceptible population by reinforcing health education, especially in nursery, kindergarten and primary school. Similarly, the prevention of syphilis also relies on health education of the public about its hazard and transmission.

Improving the surveillance system is the key to early warning of epidemics, and multivariate time series analysis could help by suggesting which variables should be included into the system and how to obtain comprehensive analysing results. On this basis, further studies with provincial level data and more variables are needed to explore the causation net of epidemics for faster and better control and prevention.
